# Amino Acids Transitioning of 2009 H1N1pdm in Taiwan from 2009 to 2011

**DOI:** 10.1371/journal.pone.0045946

**Published:** 2012-09-24

**Authors:** Guang-Wu Chen, Kuo-Chien Tsao, Chung-Guei Huang, Yu-Nong Gong, Shih-Cheng Chang, Yi-Chun Liu, Hsiao-Han Wu, Shu-Li Yang, Tzou-Yien Lin, Yhu-Chering Huang, Shin-Ru Shih

**Affiliations:** 1 Department of Computer Science and Information Engineering, Chang Gung University, Taoyuan, Taiwan, Republic of China; 2 Research Center for Emerging Viral Infections, Chang Gung University, Taoyuan, Taiwan, Republic of China; 3 Department of Medical Biotechnology and Laboratory Science, Chang Gung University, Taoyuan, Taiwan, Republic of China; 4 College of Medicine, Chang Gung University, Taoyuan, Taiwan, Republic of China; 5 Graduate Institute of Electrical Engineering, Chang Gung University, Taoyuan, Taiwan, Republic of China; 6 Department of Laboratory Medicine, Chang Gung Memorial Hospital, Linkou, Taoyuan, Taiwan, Republic of China; 7 Department of Pediatrics, Chang Gung Memorial Hospital, Linkou, Taoyuan, Taiwan, Republic of China; The University of Hong Kong, China

## Abstract

A swine-origin influenza A was detected in April 2009 and soon became the 2009 H1N1 pandemic strain (H1N1pdm). The current study revealed the genetic diversity of H1N1pdm, based on 77 and 70 isolates which we collected, respectively, during the 2009/2010 and 2010/2011 influenza seasons in Taiwan. We focused on tracking the amino acid transitioning of hemagglutinin (HA) and neuraminidase (NA) genes in the early diversification of the virus and compared them with H1N1pdm strains reported worldwide. We identified newly emerged mutation markers based on A/California/04/2009, described how these markers shifted from the first H1N1pdm season to the one that immediately followed, and discussed how these observations may relate to antigenicity, receptor-binding, and drug susceptibility. It was found that the amino acid mutation rates of H1N1pdm were elevated, from 9.29×10^−3^ substitutions per site in the first season to 1.46×10^−2^ in the second season in HA, and from 5.23×10^−3^ to 1.10×10^−2^ in NA. Many mutation markers were newly detected in the second season, including 11 in HA and 8 in NA, and some were found having statistical correlation to disease severity. There were five noticeable HA mutations made to antigenic sites. No significant titer changes, however, were detected based on hemagglutination inhibition tests. Only one isolate with H275Y mutation known to reduce susceptibility to NA inhibitors was detected. As limited Taiwanese H1N1pdm viruses were isolated after our sampling period, we gathered 8,876 HA and 6,017 NA H1N1pdm sequences up to April 2012 from NCBI to follow up the dynamics of mentioned HA mutations. While some mutations described in this study seemed to either settle in or die out in the 2011–2012 season, a number of them still showed signs of transitioning, prompting the importance of continuous monitoring of this virus for more seasons to come.

## Introduction

A swine-origin influenza A virus (S-OIV) was first found in North America in April 2009 [1] and soon became the 2009 H1N1 pandemic strain (H1N1pdm). This novel virus has been identified as a re-assortment of previously known human, avian, and swine influenza A viruses, following a complete deciphering of its 8 segmented RNA fragments [2]. In August 2010 the World Health Organization (WHO) announced that H1N1pdm infection had moved into the post-pandemic period, and predicted that localized outbreaks of various magnitudes were likely to occur for a few years, which would resemble the behavior of a seasonal influenza virus (WHO Media centre – H1N1 in post-pandemic period. 10 August 2010). While H1N1pdm was still globally seen in 2010/2011 season, the number of isolates declined considerably from its debut in 2009 season. Recent WHO reports indicated influenza A(H3N2) as the most detected virus in the northern hemisphere in 2011/2012 season. The number of global H1N1pdm cases continued to go down from its previous two seasons (less than 10% of all positive specimens for influenza), and was only found dominating in Mexico and central America (WHO FluNet, 27 April 2012).

Influenza hemagglutinin (HA) is a major antigenic glycoprotein responsible for binding the virus to the cell that is being infected. Influenza neuraminidase (NA) is another viral glycoprotein which cleaves the glycosidic linkages of neuraminic acids to free the newly formed virions away from the host cell receptors. NA is also an important drug target for the prevention of influenza infection. This is especially true because the other influenza matrix protein, M2, has evolved to significantly lose its susceptibility to adamantanes (including amantadine and rimantadine) that has been used to treat the disease for more than 30 years [3], [4]. Neuraminidase inhibitors (NAIs), including oseltamivir (Tamiflu) and zanamivir (Relenza), are the other class of antivirals used to control influenza infection. Recent reports, however, have shown that oseltamivir-resistant seasonal H1N1 viruses became widespread since the 2007/2008 season in the northern hemisphere [5].

The 2009 H1N1pdm virus acquired its HA gene directly from the classic swine influenza A virus of North American lineage, which can be further traced back to the 1918 virus [6]. The virus took its NA and M genes from Eurasian swine, which equipped it with a completely different set of NA and M genes from those of seasonal H1N1 or H3N2–which are reportedly resistant to the above-mentioned antivirals at different levels. Thus far all H1N1pdm viruses, unfortunately, have been found to be resistant to amantadine and remantadine (WHO fourth NIC Meeting Report in the Western pacific Region, May, 2010). Although most of the tested H1N1pdm viruses at the end of 2009/2010 season were still susceptible to zanamivir and oseltamir, rare cases were shown to share a single amino acid substitution H275Y in their NA gene, which costs drug susceptibility [7]. More than one study also indicated that the excessive use of NAI drugs is likely to increase the chance of NAI-resistant viruses evolving [8], [9].

The current study elucidated the evolutionary dynamics of H1N1pdm, based on 77 and 70 isolates which we collected, respectively, during the 2009/2010 and 2010/2011 influenza seasons in Taiwan. It was found that the amino acid mutation rates for both HA and NA nearly doubled in the second season than they were in the first season. In particular that some of the newly found mutation markers in the second season showed statistical correlation to disease severity. Although there were five noticeable HA mutations made to antigenic sites, no visible titer changes were detected based on hemagglutination inhibition tests. All Taiwanese isolates maintained susceptibility to NAIs, except one isolate observed with drug resistance marker H275Y in early 2011.

**Figure 1:**
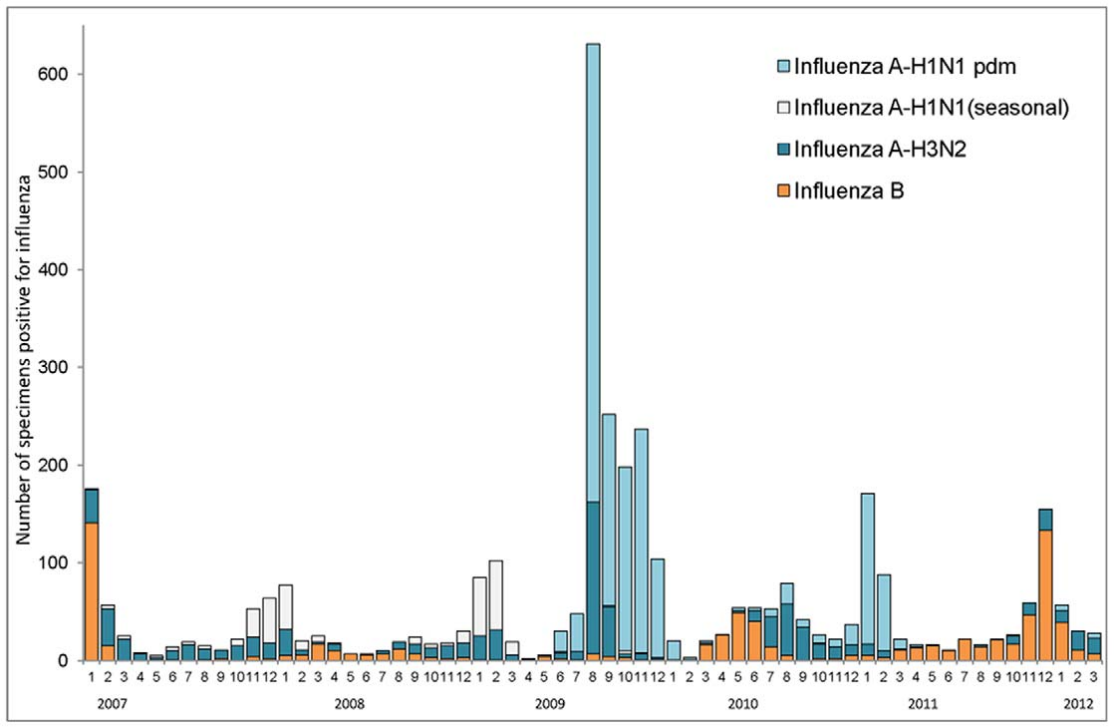
Chronological surveillance of influenza viruses in Chang Gung Memorial Hospital from 2007 to 2012.

## Materials and Methods

### Ethics Statement

This study has been approved by the Institutional Review Board (IRB) of Chang Gung Medical Foundation, Linkou Medical Center, Taoyuan, Taiwan. The IRB approved number is “98-2707B” and the topic is titled “Antiviral Susceptibility Surveillance of Novel Swine-Origin Influenza A H1N1”. We used residual virus isolates grown from the specimens of potential influenza A H1N1 patients during their routine checkup. Since no extra clinical specimens were collected from patients and human specimens were not directly used in this research, the IRB agreed that no written or verbal informed consent was necessary.

### Sample Collection

We collected 1,590 H1N1pdm isolates at Chang Gung Memorial Hospital (CGMH), Taoyuan, Taiwan, from June 2009 to February 2011. Two to four of these isolates per week were recruited and their HA and NA sequences were produced and analyzed. A total of 147 samples were obtained, including 77 from June 2009 to May 2010 in the first pandemic season, and another 70 from August 2010 to February 2011 in the second season.

**Table 1:** Monthly HA gene mutation frequencies (in percentage) for H1N1pdm.

Mutation	Type	2009/10										2010/11							Total (%)
		Jun	Jul	Aug	Sep	Oct	Nov	Dec	Jan	Feb	May	Aug	Sep	Oct	Nov	Dec	Jan	Feb	
L8M	II.b													14	17			30	5.4
T14I	II.c																17	30	6.1
P100S	I.a	100	100	100	100	100	100	100	100	100	100	83	100	100	100	100	100	95	98.6
D114N	II.c																28	45	9.5
N142D	II.a										50	50	60	43	83	25	11		12.9
S160G	II.b													29		25	50	40	14.3
S202T	II.b													43	17	63	72	65	23.8
T214A	I.b	92	100	100	100	100	100	100	100	100	100	100	100	57	100	50	50	55	82.3
S220T	I.a	92	100	86	100	89	89	100	100	100	50	100	100	100	100	100	100	90	95.2
R222K	I.c			14													17	30	6.8
I233V	II.c																17	35	6.8
V266L	I.c				7	11											28	35	9.5
K300E	I.c	8															11	30	6.1
I338V	I.a	100	100	100	93	100	100	100	100	100	100	100	100	100	100	75	100	95	97.3
E391K	I.b	8		29	43	78	75	100	75	100	50	67	60	86	100	75	33	80	59.2
S468N	I.b	8					13							43	17	63	72	65	25.2
Samples/month		13	5	7	14	9	8	9	8	2	2	6	5	7	6	8	18	20	147

### Statistical Methods

Student’s *t*-test was used to assess the overall difference in terms of mutation frequencies between severe (pneumonia, acute respiratory distress syndrome, or expired) and non-severe (other upper respiratory tract infections) cases of H1N1pdm infections. The correlation of site-specific mutations between severe and non-severe cases was evaluated using two-tailed Fisher’s exact test.

### Nucleotide Sequencing

Nucleotide sequences of HA and NA were obtained using RT-PCR and the Sanger dideoxy sequencing method. A total of six primer pairs were used for sequencing HA and NA genes, and are listed in Table S1. The obtained amplicons were assembled into a full-length 1,701-bp span for HA and 1,410-bp for NA, using DNASTAR Lasergene (DNAStar, Madison, WI).

**Table 2:** Monthly NA gene mutation frequencies (in percentage) for H1N1pdm.

Mutation	Type	2009/10										2010/11							Total (%)
		Jun	Jul	Aug	Sep	Oct	Nov	Dec	Jan	Feb	May	Aug	Sep	Oct	Nov	Dec	Jan	Feb	
M15I	II.a										50	17	40	29	67		11		8.2
N44S	II.b													43	17	75	50	45	19.0
V106I	I	100	100	100	100	100	100	100	100	100	50	100	100	100	100	100	100	100	99.3
N189S	II.a											17	60	29	67		11		8.2
V241I	II.b													43	17	88	72	60	24.5
N248D	I	100	100	100	100	100	100	100	100	100	100	100	100	100	100	100	100	100	100
S299A	II.c																17	35	6.8
I365T	II.a											33	20	29	50				5.4
N369K	II.b													43	17	88	72	65	25.2
I374V	II.c																17	35	6.8
Samples/month		13	5	7	14	9	8	9	8	2	2	6	5	7	6	8	18	20	147

### Sequence Analysis

Nucleotide sequences were aligned and translated into protein sequences using BioEdit (Tom Hall, Ibis Biosciences, Carlsbad, CA). The prototype strain A/California/04/2009-the first H1N1pdm isolate and candidate vaccine virus used by the Centers for Disease Control and Prevention (CDC/USA), served as a reference strain to display the amino acid changes in the investigated Taiwanese strains. GenBank accession numbers, for HA and NA of A/California/04/2009, are FJ966082 and FJ966084, respectively.

### Nucleotide Sequence Accession Numbers

All newly-reported sequences in this study have been deposited at GenBank database under the accession numbers CY045226, CY045234, CY045242, CY047744, CY053474, CY053482, CY053490, CY053498, CY053506, and JN381203-JN381340 for the HA genes; and CY045228, CY045236, CY045244, CY047746, CY053476, CY053484, CY053492, CY053500, CY053508, and JN381343-JN381480 for the NA genes.

**Table 3:** Monthly accumulated amino acid mutation counts and frequencies (mutations per sequence) for 147 H1N1pdm HA and NA genes isolated in Chang Gung Memorial Hospital, Taiwan.

		HA		NA	
	Seq cnt	Accu. mut.	Mut. freq.	Accu. mut.	Mut. freq.
Jun/2009	13	59	4.54	29	2.23
Jul	5	21	4.20	11	2.20
Aug	7	35	5.00	19	2.71
Sep	14	73	5.21	33	2.36
Oct	9	50	5.56	21	2.33
Nov	8	44	5.50	20	2.50
Dec	9	50	5.56	22	2.44
Jan/2010	8	47	5.88	22	2.75
Feb	2	14	7.00	5	2.50
May	2	12	6.00	7	3.50
Aug	6	45	7.50	21	3.50
Sep	5	39	7.80	24	4.80
Oct	7	57	8.14	35	5.00
Nov	6	54	9.00	35	5.83
Dec	8	65	8.13	48	6.00
Jan/2011	18	138	7.67	97	5.39
Feb	20	180	9.00	101	5.05
Season 1	77	405	**5.24** a	189	**2.45** b
Season 2	70	578	**8.26** c	361	**5.16** d
Total	147	983	6.69	550	3.74

a 9.29×10^−3^,

b 5.23×10^−3^,

c 1.46×10^−2^,

d 1.10×10^−2^ substitutions per amino acid site.

## Results

### Influenza Epidemiology in Taiwan

Figure 1 shows the chronological surveillance of influenza viruses at Chang Gung Memorial Hospital (CGMH) in Taiwan from 2007 to 2012. Although influenza B appeared to dominate in the winter of 2006/2007, positive cases of seasonal H1 viruses rose during the following two winters. During the 2009 H1N1pdm outbreak, large number of H1N1pdm cases were detected between approximately June 2009 and January 2010, similar to what was observed elsewhere in the world. H1N1 pdm activity seemed to die out after its historical debut, and influenza B viruses took over in the first half of 2010, followed by predominantly influenza H3 viruses in the summer months. Sporadic H1N1pdm cases were still observed in the summer months of 2010, until a surge of H1N1pdm cases emerged from mid- to late 2010 until early 2011. Influenza B viruses were mostly detected since April 2011, and peaked in the winter months of 2011/2012. Some influenza A H3N2 cases were also observed, with very limited H1N1pdm viruses detected in the 2011/2012 season. In the discussion that follows, for the sake of convenience we define the first H1N1pdm season as having spanned from June 2009 to May 2010, and the second season from August 2010 to February 2011. These time periods were ascertained from the distribution of isolation counts, shown in Figure 1.

**Table 4:** Correlation of mutation frequencies on HA and NA genes between severe and non-severe cases of H1N1pdm infection.

	HA		NA	
	Mutation frequency	*p-*value*	Mutation frequency	*p-*value*
Non-severe cases^a^ (N = 82)	6.1	–	3.3	–
Severe cases (N = 51)	7.1	0.001	4.4	<0.001
Pneumonia (N = 37)	6.7	0.09	4.3	<0.001
ARDS^b^ or Expired (N = 14)	8.5	<0.001	4.6	<0.05

a Upper respiratory tract infection.

b Acute respiratory distress syndrome.

* Student’s *t*-test.

**Table 5:** Correlation of significant mutation sites on HA and NA genes between severe and non-severe cases of H1N1pdm infection.

	HA (%)				NA (%)			
	S160G	S202T	V266L	S468N	N44S	V241I	S299A	N369K
Non-severe cases^a^(N = 82)	8(9.7)	14(17)	2(2.4)	16(19.5)	0 (0)	13 (16.0)	0 (0)	13 (16.0)
Severe cases^b^(N = 51)	12(23.5)	21(41.2)	9(17.6)	21(41.2)	15 (29.4)	21 (41.2)	4 (7.8)	21 (41.2)
*p-*value*	0.04	0.003	0.003	0.009	<0.0001	0.002	0.02	0.002

a Upper respiratory tract infection.

b Pneumonia, Acute respiratory distress syndrome, Expired.

* Fisher’s exact test.

### Amino Acid Transitioning of Taiwanese H1N1pdm Viruses

All sequences were compared to A/California/04/2009 to highlight their amino acid changes. Among the 566 amino acid positions of the full-length HA gene in 147 Taiwanese H1N1pdm strains (spanning the two influenza seasons from June 2009 to February 2011), 100 positions (17.7%) were found with amino acid substitutions. Although many of these changes were merely transient, 16 positions showed a mutation frequency of more than 5% among the 147 HA samples. Table 1 lists their monthly mutation frequencies; March, April, June, and July of 2010 were excluded as no H1N1pdm viruses were isolated in CGMH during those months. It is clear that P100S (98.6%), S220T (95.2%), and I338V (97.3%) dominated since June 2009, and still maintained over 90% frequencies in season-ending months in 2011. These three mutations quickly settled and dominated in the virus population were assigned to type I.a in Table 1. T214A was also frequently seen throughout the entire sampling time, yet it appeared in only approximately half the viruses isolated from December 2010 to February 2011. E391K was another substitution that was less seen at the beginning of pandemic but largely emerged only after September 2009. It resembled the temporal distribution of T214A which appeared less frequently in season-ending months, in contrast to the total dominance observed in type I.a mutations. Both T214A and E391K were grouped as type I.b mutations, together with S468 which was sporadically seen in the first season and commonly appeared since October 2010 in the second season.

The remaining 10 signatures appeared at various stages in these two seasons. For example, three group I.c mutations were sporadically observed relatively early in 2009, including R222K in August, V266L in September/October, and K300E in June. They remained completely silent, however, for the entire 2010 and appeared again only in early 2011. Seven other group II mutations appeared only in the second season, including one type II.a mutation N142D since the summer of 2010; three type II.b mutations L8M, S160G and S202T since October 2010; and three type II.c mutations T14I, D114N and I233V since January 2011. Interestingly, T14I, D144N, R222K, I233V, V266L, and K300E seemed to have a synchronized appearance in January and February of 2011, either for the first time or as a re-emergence after being absent for the entire 2010. All 16 HA mutations still showed at least 30% frequency (6 out of 20 cases) in February 2011, except for N142D which was last seen in only two out of 18 viruses in January 2011 but none in February. The complete amino acid mutation statistics of 147 Taiwanese HA sequences can be seen in Table S2.

NA protein sequences of 469-aa for the same 147 Taiwanese H1N1pdm strains were also analyzed. We found 69 positions (14.7%) that had different amino acid residues from A/California/04/2009. Many of these NA mutations were rarely seen, similar to what was observed in HA. Table 2 shows ten major NA sites with more than 5% mutation frequency, among which V106I and N248D of type I were present at all times. Other eight mutations entered no earlier than May 2010, with three type II.a mutations M15I, N189S, and I365T ceasing to appear in the final months of the 2010/11 season. In other words, only five new primary NA mutations survived at the end of February 2011; of these, three type II.b mutations N44S, V241I, and N369K had not been seen until October 2010, and two type II.c mutations S299A and I374V were only observed after January 2011. Interestingly, the appearance of NA mutations of type II.a, II.b and II.c completely synchronized with type II.a, II.b and II.c HA mutations listed in Table 1, respectively. The complete amino acid mutation statistics of the 147 Taiwanese NA sequences can be seen in Table S3.

**Table 6:** NA amino acid substitutions and their frequencies (mutation count divided by total count) in drug-associated sites of Taiwanese H1N1pdm viruses.

Month/Residue/Pos	I223	S247	H275	N295
Jun/2009				
Jul				
Aug				
Sept				
Oct				
Nov				
Dec				
Jan/2010	T(1/8)			
Feb				
May				
Aug				
Sept				
Oct				
Nov				
Dec		N(1/8)		
Jan/2011				K(2/18)
Feb			Y(1/20)	

Table 3 shows the statistics of amino acid substitutions for the two H1N1pdm seasons investigated. The mutation counts shown here represent all amino acid substitutions based on A/California/04/2009 within the given month. A mutation frequency was computed by dividing the accumulated mutations by the sample count per month, per season, and so on. The mutation frequencies were higher for HA than for NA protein in all months. We also found that the mutation frequencies in the second season were 8.26 sites per 566-aa HA sequence (or 1.46×10^−2^ per amino acid site) and 5.16 sites per 469-aa NA sequence (or 1.10×10^−2^ per amino acid site). These rates were elevated compared with the 5.24 and 2.45 sites per sequence for HA and NA (or 9.29×10^−3^ and 5.23×10^−3^ per amino acid site), respectively, found for the previous season–that is, when the pandemic first took place in 2009.

**Table 7:** Various HA mutations in the first H1N1pdm season from four different geographical locations.

	Time	Cnt	K2E	I4T		F12L		A13V		A15T			K39R	V47A		D52N		P100S		S101N
TW	6∼9/2009	39				1		1					3	1		3		**39**		1
IND	5∼9/2009	13	2	2						1								**13**		
RUS	∼1/2010	23																**23**		
CAN	5∼12/2009	210	**55**																	
	**Time**	**Cnt**	**D103N**	**P141S**		**S145P**		**H155Y**		**K171E**			**G172E**	**S179N**		**A212G**		**T214A**		**S220T**
TW	6∼9/2009	39		1				1										**38**		**37**
IND	5∼9/2009	13								1								**13**		**10**
RUS	∼1/2010	23	1			7							1	1		1		**23**		**21**
CAN	5∼12/2009	210																		**72**
	**Time**	**Cnt**	**R222K**	**K226E**		**K228E**		**D239G/E**		**Q240R**			**V251I**	**V266L**		**N277D**		**K300N/E**		**Q310H**
TW	6∼9/2009	39	1					1E						1				1E		
IND	5∼9/2009	13						1G		1			1							2
RUS	∼1/2010	23		1		1		6G, 1E		2			1			3		1N		1
CAN	5∼12/2009	210	6					2G, 3E												**55**
	**Time**	**Cnt**	**P314S**	**P321S**		**I338V**		**S343Y**		**G356R**			**D381G**	**N387K**		**E391K**		**K419T**		**V428I**
TW	6∼9/2009	39	1	1		**38**		1					2			9				1
IND	5∼9/2009	13				**13**				1				1						
RUS	∼1/2010	23	1			**21**														
CAN	5∼12/2009	210														8		**37**		
	**Time**	**Cnt**	**N458D**		**N461K**		**S468N**		**I477T**		**K521N**	**R526K**			**I527T**		**I564T**			
TW	6∼9/2009	39			1		1		1		1									
IND	5∼9/2009	13					3										3			
RUS	∼1/2010	23	1												1					
CAN	5∼12/2009	210										6								

TW: Taiwan; IND: India; RUS: Russia; CAN: Canada.

We further traced 133 samples whose clinical records are available and compared their disease severity with the observed HA/NA diversity. Among these, 82 are non-severe cases of upper respiratory tract infection and 51 are severe cases of lower respiratory tract infection, including 37 with pneumonia and 14 with acute respiratory distress syndrome (ARDS) or expired. As shown in Table 4, viruses from patients that were very sick did seem to mutate more. Table 5 lists a number of amino acid sites that exhibit significant difference in mutation frequencies between the non-severe and severe cases. Interestingly, all these eight signatures emerged only in second season (no earlier than October of 2010), suggesting their roles in complicating the disease as the virus evolved.

### HA Antigenicity and Receptor Binding

Igarashi et al. [10] predicted a number of antigenic sites of H1N1pdm hemagglutinin by homology modeling of the earlier H1N1 viruses. Approximately half of these antigenic residues (7 of 13 Sa sites, 6 of 12 Sb sites, and 8 of 19 Ca sites) showed amino acid substitutions in the investigated Taiwanese HA sequences; an exception was the Cb site, for which all six antigenic residues remained unchanged. Many of these substitutions, however, occurred in rather limited cases. For example, some substitutions occurred only once among 77 Taiwanese viruses collected in the first pdm season, and were no longer observed at all during the second season. These substitutions included P141S and K177T in Sa, A203T and A212V in Sb, and H155Y and I183F in Ca. Others emerged only in the second season, typically only once but at most twice; these substitutions included G172E, N173T, P176S, and K180T in Sa; T201N, S207I, and L208I in Sb; and G187R in Ca. Some transient substitutions were found in Ca sites for both seasons. For example, A158S and D239E were found in the first season, whereas in the second season they were changed into different residues of E and G, respectively. Aside from these rare substitutions, S220T in Ca was found to dominate in all months of both seasons, as shown in Table 1. Antigenic mutations seen mainly in the second season included N142D in Sa and S202T in Sb, although the former seemed to die out after December 2010 and had completely disappeared by February 2011 (Table 1). In contrast, S202T has recently become dominant in Taiwan only in end-of-season months. The last two noticeable antigenic mutations included A156T and R222K in Ca. Mutation A156T occurred only in four of 13 viruses collected from October to November 2010, and went completely silent for the remainder of the season. Mutation R222K was first seen only in August 2009, and re-emerged in nine out of 38 viruses collected in January and February 2011. Details of the described antigenic mutations can be seen in panels 1 to 4 of Table S4.

**Figure 2:**
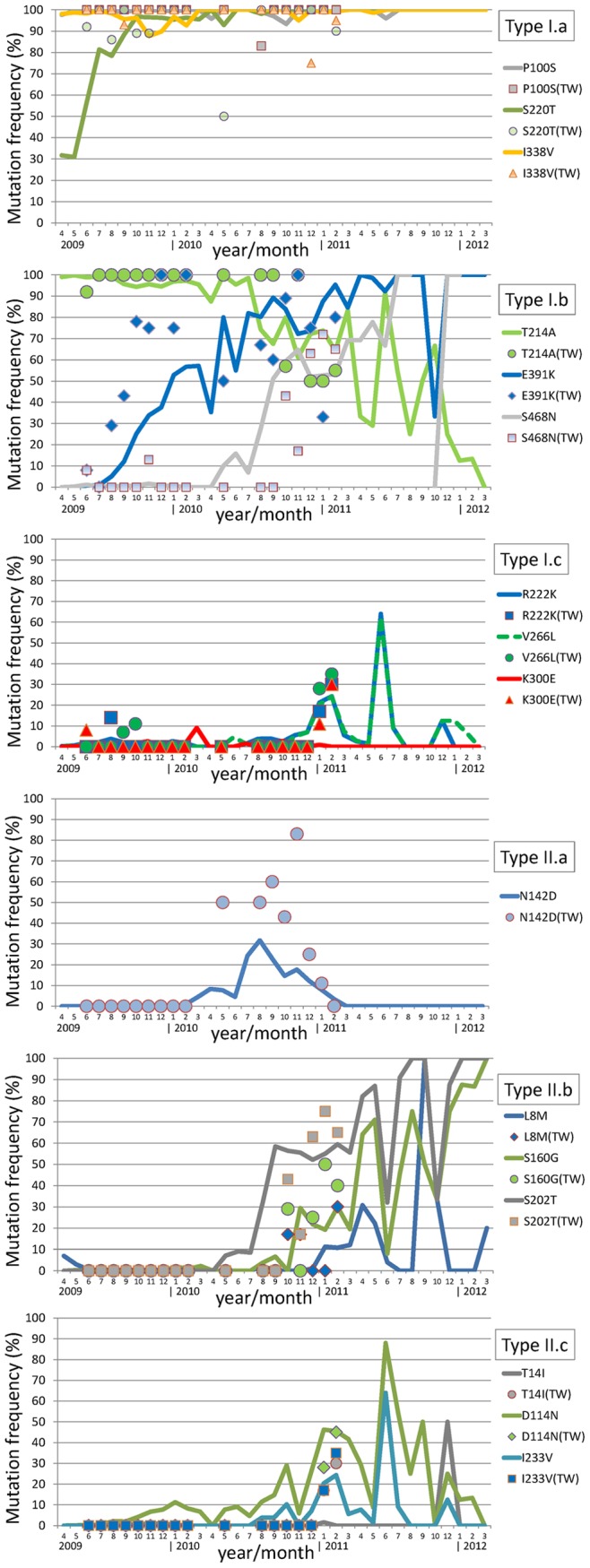
HA mutation dynamics of Taiwanese H1N1pdm viruses versus publicly available H1N1pdm viruses. Horizontal axis represents the year/month the sampling took place, and the vertical axis represents the frequency (in percentage) that one particular mutation occurred in the month. Taiwanese data range from June 2009 to February 2011, and are graphed by various markers (circles, squares, triangles, and diamonds). A total of 8,876 H1N1pdm HA sequences are collected from NCBI which cover a 3-year span from April 2009 to March 2012 (no case in April 2012), and are graphed in thick lines. Mutations are grouped according to the transitioning types described in Table 1, and are displayed from top to bottom as type I.a, I.b, I.c, II.a, II.b and II.c. Note that some lines are broken due to that no samples are found in these months. Various lines may have different breakpoints since some NCBI sequences are partial, making total positional counts vary. For example, there is no amino acid 468 (shown in a gray line in the second subpanel) available in September 2011, as well as in February and March of 2012.

**Figure 3:**
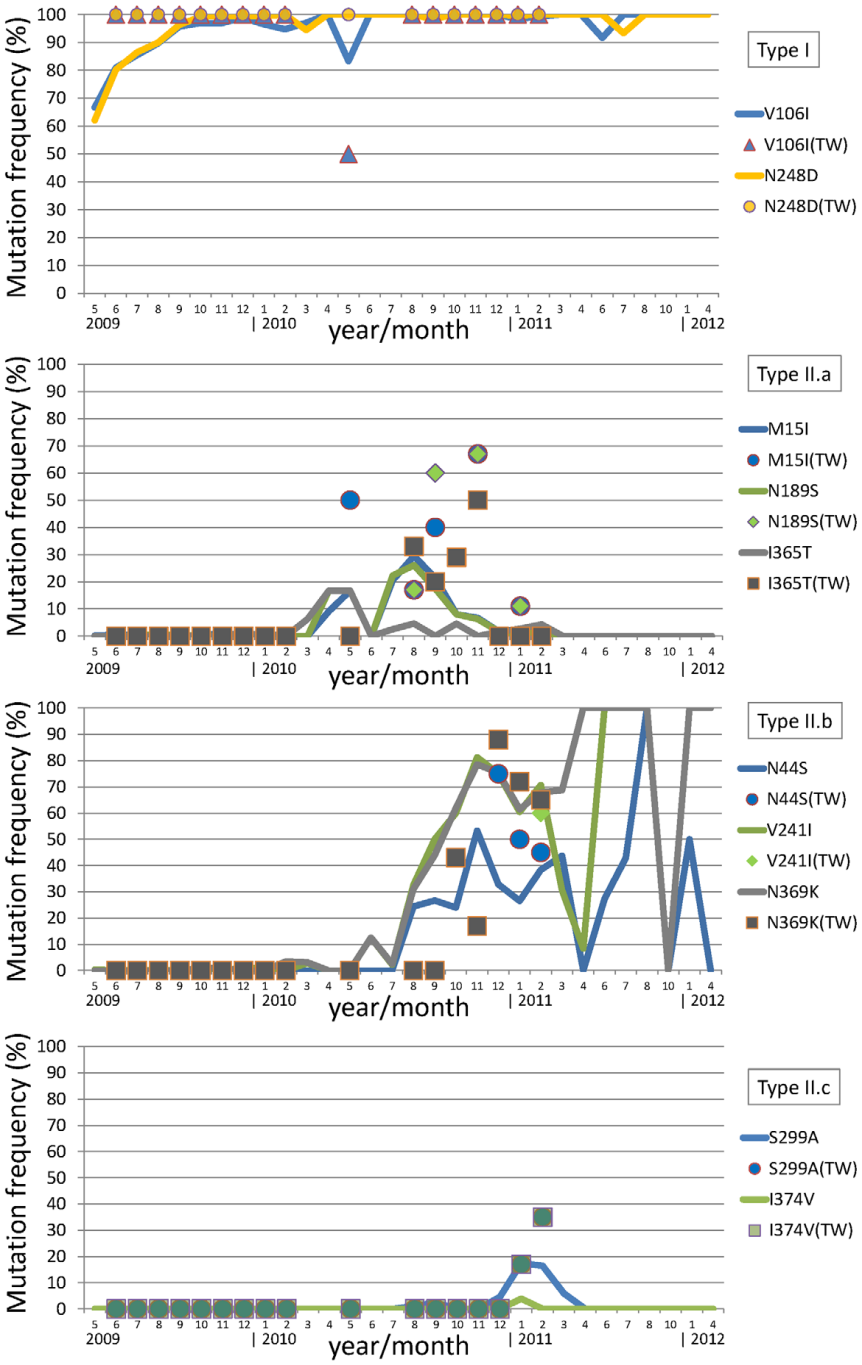
NA mutation dynamics of Taiwanese H1N1pdm viruses versus publicly available H1N1pdm viruses. Horizontal axis represents the year/month the sampling took place, and the vertical axis represents the frequency (in percentage) that one particular mutation occurred in the month. Taiwanese data range from June 2009 to February 2011, and are graphed by various markers (circles, squares, triangles, and diamonds). A total of 6,017 H1N1pdm NA sequences are collected from NCBI which cover a 3-year span from April 2009 to April 2012, and are graphed in thick lines. Mutations are grouped according to the transitioning types described in Table 2, and are displayed from top to bottom as type I, II.a, II.b and II.c.

Following the observation that a number of HA amino acid changes were located on the predicted antigenic sites, hemagglutination inhibition (HI) tests were additionally performed to assess if the mutated viruses have changed their antigenic profile. Ferret anti-serum against A/California/07/2009 (kindly provided by Dr. Ming-Tsan Liu, Taiwanese CDC) was used in these tests. It was mentioned earlier that there are five HA locations with noticeable amino acid changes in the predicted antigenic sites, including A156T, S220T and R222K in Ca, D142D in Sa and S202T in Sb. We therefore selected nine viruses (all in winter season of 2010/2011) each contains at least two of these five transitions and found their HI titers ranging from 1∶1280 to 1∶5120, suggesting no antigenic change for the investigated Taiwanese H1N1pdm viruses regardless that many HA mutations were observed (data not shown).

Yang et al. [11] listed HA 148∼152 (the 130-loop), 201∼208 (the 190-helix), and 235∼242 (the 220-loop) as the receptor-binding sites (RBS) of the 2009 H1N1pdm viruses. Seven RBS positions displayed amino acid changes in the investigated Taiwanese strains. Six of them were also found in antigenic sites described in the previous paragraphs, including T201N, S202T, A203T, S207I, and L208I in Sb, and D239G/E in Ca. The only non-antigenic RBS mutation was A151T, with only one out of five cases reported in September and one of eight cases in December 2010 during the second Taiwanese H1N1pdm season. Details of the RBS mutations can be seen in panel 5 of Table S4.

### NA Antigenicity

NA antigenicity was less studied in the past than was HA antigenicity. Maurer-Stroh et al. [12] identified a number of NA antigenic regions of the new H1N1pdm via a homology-based 3D structure modeling and epitopes mapping. These regions included positions 83∼99, 103∼144, 156∼190, 252∼303, 330, 332, 340∼345, 368, 370, 386∼395, 400, 431∼435, and 448∼468. Among these 193 predicted NA antigenic sites, only 31 (16.1%) showed amino acid changes in our collected samples, compared with 21 out of 50 (42.0%) of the HA antigenic sites that showed variations. Similar to what has been observed in mutating HA antigenic sites, most of the NA antigenic mutations identified were found to occur only rarely. Details of the described antigenic mutations can be seen in panel 6 of Table S4.

### NA Genetic Diversity and Susceptibility to NAI

Several amino acid substitutions of influenza A virus are known to confer resistance to NAI [13]–[18]. These include E119V, D151N, S247N, H275Y and N295S, among which H275Y has been used as a primary marker for diagnosing resistant viruses. Recent homology modeling for the NA gene of the new H1N1pdm also identified additional 13 NA residues (118, 152, 156, 179, 180, 223, 225, 228, 277, 278, 293, 368, and 402) that were important for contacting sialyloligosaccharide substrates directly, participating in catalysis, or providing a structural framework to the bound drug [12]. Table 6 summarizes the above-mentioned drug-associated sites that showed mutations in our sequenced Taiwanese NA gene. Only four mutations were found in a limited count of viruses. In particular, only one (A/Taiwan/90252/2011) of 20 isolates was found with H275Y in February 2011. Other than H275Y, two more mutations occurred in late months of the second season, including one S247N in December 2010 and two isolates with N295K in January 2011. Note that the two mutations in NA 295 in Taiwan were converted into K rather than into S; the latter has been reported to affect susceptibility to NAIs in seasonal viruses [13], [17]. The last drug-associated mutation I223T appeared earlier in January 2010 at the wake of the first H1N1pdm season which structural modeling has predicted as a drug binding site [12].

## Discussion

### Evolution of H1N1pdm HA Gene in Taiwan

In this work we analyzed 147 Taiwanese H1N1pdm viruses to portray the evolutionary dynamics of its historical debut. We collected 77 samples for the 2009/2010 season and 70 samples for the 2010/2011 season, and we divided the two seasons by including the two cases in May of 2010 into the first season. Because no data were collected for March and April 2010 and for June and July 2010, the two May cases might just as well have been assigned to the beginning of the second season instead. The overall statistics discussed here, however, would most likely not have been affected either way. We detected 51 HA sites showing changes in the first pdm season in Taiwan. Moving into the second pdm season, only 22 of the 51 HA mutations had maintained such changes. The other 29 HA sites were found to have recovered their amino acids to what was originally observed in A/California/04/2009. Nevertheless, 49 new HA sites showed amino acid substitutions that had not been observed in the first pdm season, bringing the number of HA sites showing amino acid changes in the second season (relative to the original 2009 California strain) to 71.

Pan et al. [19] analyzed H1N1pdm strains deposited to GenBank between April 1 and December 31, 2009, reported only one dominant HA mutation S220T and described its location near the receptor-binding domain (RBD). They did not observe the other four major HA mutations that we have reported in Table 1. Potdar et al. [20] reported an Indian study involving 13 H1N1pdm isolates from May to September 2009. They found the HA sequences dominated by four mutations, namely P100S, T214A, S220T, and I338V. These results are consistent with our findings, except that Potdar et al. did not notice any E391K mutation. Despite approximately the same time-frame between the two studies (May-September 2009 for India, and June-September 2009 for Taiwan), only six mutations were found in common between 16 mutations detected from 13 Indian viruses and 26 mutations from 39 Taiwanese viruses (shown in Table 7). Ilyicheva et al. [21] performed a sequence analysis of 23 Russian H1N1pdm strains and reported 20 HA mutation sites. Graham et al. [22] performed HA sequence analysis of 235 Canadian H1N1pdm strains, of which 210 were collected from May to December 2009. Taking together the Indian and Taiwanese cases spanning approximately the same period of time in the first H1N1pdm season, these mutations were detected from viruses isolated in four distinct geographical locations, representing an H1N1pdm HA mutation spectrum around the globe. Most of the sporadic mutations did not occur at sites that are common across these four locations, suggesting that a geographical variation did exist in the early diversification of the viral genome.

All primary HA mutations observed in the first Taiwanese H1N1pdm season remained abundant in the second season. These include P100S, S220T and I338V. Although T214A substitution was also found dominated throughout the two seasons, its appearance was less frequent (∼50%) in the end-of-season months December 2010 to February 2011. Table 1 also notes other dynamic patterns of HA mutations, including the early emerging and re-emerging mutations of types I.b and I.c, and second-season emerging types II.a, IIb and II.c. The possible association between these dynamic patterns and HA evolution, particularly at a stage after the so-called early diversification of this new virus, is an interesting question to address. Many of these mutations were still present with over 50% occurrence at the season-ending month. The late-breaking mutations (T14I, D114N and I233V of type II.c, and R222K, V266L and K300E of type I.c) were seen at approximately the same frequencies of 11–28% in January 2011, which had nearly doubled to 30–45% in February 2011. Although their prevalence was not as obvious as that of other mutations, our data suggest they may re-appear in the subsequent seasons.

As limited Taiwanese H1N1pdm viruses were isolated and investigated after our sampling period, we gathered 8,876 H1N1pdm HA sequences up to April 2012 from National Center for Biotechnology Information (NCBI) and analyzed the dynamics of mentioned HA mutations. As shown in Figure 2, the temporal appearance of T214A was in general declining yet in an oscillatory manner after March 2011. For example, T214A was only seen in 2 out of 15 HA sequences in February 2012, and none among 5 cases in March 2012. Figure 2 also supports our finding for the two late emerging/re-emerging type II.c mutations D114N and I233V, and two type I.c mutations R222K and V266L. Their dynamic patterns after March 2011 seem to resemble what was observed in T214A. Interestingly, the remaining type I.c mutation K300E and type II.c mutation T14I are hardly seen in these NCBI HA sequences. In contrast to the generally declining T214A, the other two type I.b mutations E391K and S468N and two type II.b mutations S160G and S202T after the second season appear to be stabilizing in the virus population. Continuous monitoring these mutations in more seasons to come is important to better understand the HA evolutionary spectrum of this virus.

### Evolution of H1N1pdm NA Gene in Taiwan

We mentioned that only 22 out of 51 HA mutations (43.1%) detected in the first season showed up again at least once in the second pdm season in Taiwan. Of all 71 HA mutations observed in the second season, 49 mutations (69.0%) had not appeared at all in the first season. Such diversification for giving up old and acquiring new mutations across seasons was even more noticeable for NA, in which only 25% of the mutations (7 out of 28) from the first season survived in the second season, and 85.4% (41 out of 48) of the mutations found in the second season were newly emerged. A number of the newly emerged second-season mutations were short lived and had disappeared completely in the final months of the season, including the three type II.a mutations M15I, N189S, and I365T. The three type II.b mutations N44S, V241I, and N369K began in October 2010 and appeared to persist until the season’s end, although they never reached 100% peak as did V106I and N248D. Recall that the previously mentioned HA mutations of L8M, S160G, S202T, and S468N were also found to emerge or re-emerge in October 2010. Nevertheless, similar to a number of type I.c and type II.c HA mutations observed in the final two months of the second season (T14I, D114N, R222K, I233V, V266L and K300E), we also found that the two type II.c NA mutations S299A and I374V emerged only in January and February 2011. Such co-incidence in the evolutionary dynamics of HA and NA suggests that a fitness or co-evolution occurs between the two genes, which may play an important role in shaping the viral genome for many seasons to come.

We gathered 6,017 H1N1pdm NA sequences from NCBI to follow up those mentioned NA mutations in Taiwan. Figure 3 describes their temporal dynamics for the entire three H1N1pdm seasons from April 2009 to April 2012. Other than that V106I and N248D are fixed in the population as expected, those three type II.b mutations (N44S, V241I and N369K) that showed higher prevalence in 2010–2011 season seem to appear in a oscillatory manner. Note that a number of 2011–2012 months are missing in Figure 3 because of no NA sequences are available in NCBI. Nevertheless, the sample counts of the final four months in Figure 3 (August/October of 2011, and January/April of 2012) are only three, two, three and one, respectively. More samples are needed in order to better describe NA evolution in terms of amino acid transitioning.

### Mutation Rates of Taiwanese H1N1pdm Viruses in 2009–2011

A nationwide molecular surveillance of H1N1pdm genomes in Canada [22] revealed HA and NA genetic diversity at 1.98×10^−3^ and 2.36×10^−3^ amino acid substitution per protein site, respectively. This study’s sampling covered a period of only eight months (from the emergence of H1N1pdm to December 5, 2009) and the NA proteins seemed to display slightly more substitutions than did the HA proteins. Our rates of amino acid substitution for HA and NA in the first season (a 12-month period) were 9.29×10^−3^ and 5.23×10^−3^, respectively. To make these comparable with Canadian’s study, a sub-sampling from June to November 2009 was chosen and mutation rates were re-computed as 8.90×10^−3^ for HA and 4.68×10^−3^ for NA. Not only did our HA proteins display far more diversity than NA, our study also found much higher mutation rates.

Earlier we discussed the difference in HA mutating sites between Taiwanese and Canadian studies over the same sampling period (Table 6). We noted that P100S, T214A, S220T, and I338V occurred in almost all 39 Taiwanese H1N1pdm viruses sampled. For the 210 Canadian viruses collected within the same time period, however, only 72 (34.3%) were observed to contain S220T; no P100S, T214A, or I338V changes were found. Two other major mutations, K2E and Q310H, were detected in 55 (26.2%) of the Canadian HA proteins but not at all in Taiwanese strains. However, the overall HA mutation rate for Taiwanese viruses was apparently higher than that of the Canadian ones. Such geographical disparity may explain the differences in mutation rates described here.

Furuse et al. [23] compared the evolutionary rates among seasonal H1N1 (1918–1957 and 1977–2009) virus, swine H1 virus, and 2009 H1N1pdm virus; their results indicated that the rate of H1N1pdm was much lower than that of the others. However, the pdm data analyzed had been sampled over a period of less than ten months, up to the end of the first pandemic season. This sampling limitation may have led to an unreliable estimation of the correlation coefficient for describing the evolutionary trend. The Canadian study mentioned above was also based on an eight-month period only, and many genetic variants were still evolving, at least for the first pandemic season. Nevertheless, our second-season evolutionary statistics indicated that the amino acid mutation rates of H1N1pdm HA and NA are elevated than they were in the first H1N1pdm season. For the mutation frequencies shown in Table 3, the Taiwanese H1N1 viruses showed 5.24 substitutions per 566-aa HA segment in the first season, increasing to 8.26 (a 57.6% boost) in the second season. NA displayed a lower mutation frequency than HA in the first season, with 2.45 substitutions per 469-aa segment, but soared to 5.16 (a 111% boost) in the second season. Ongoing surveillance data obtained from various geographical locations and subsequent seasons will enable more accurate descriptions of the evolutionary dynamics of this novel H1N1pdm virus.

It is mentioned that the way these clinical samples were collected did not take into consideration the demographic factors such as gender, age or geographical location. Neither did we gather vaccination history from the patients. As a result, these data are not suitable for revealing correlations between these factors and amino acid mutations. A large-scale, island-wide study by Taiwanese CDC [24] revealed that the major affected groups were shifted to older individuals of higher age-specific case fatality ratios (CFRs) from May 2009 to April 2011 in Taiwan. They also discussed the possibility that the shift could be attributed to the vaccination program in which adults aged 18–64 were the shortfall in influenza vaccination. How such shifting of CFRs relates to underlying genome variations, however, remains to be investigated.

In summary, we revealed amino acids transitioning of the two surface glycoproteins of H1N1pdm viruses, particularly on how these mutations shifted in 2010/2011 season after the H1N1pdm’s debut in 2009/2010. We found 17.7% of HA and 14.7% of NA sites had their amino acids mutated based on A/California/4/2009. Many of these mutations were transient, demonstrating how the viral genome has been shaped dynamically. Among those mutations that appeared more frequently (>5% incidence in all 147 viruses from June 2009 to February 2011), many were new after August 2010 which were not seen throughout the first pandemic season (Tables 1 and 2). Furthermore, some late-breaking mutations are found to have statistical correlation to disease severity. Although a number of mutations were made to the antigenic sites, HI tests showed no titer changes for these Taiwanese strains. There was only one recent isolate in February 2011 which contains the well-known resistance marker H275Y in NA, suggesting an overall susceptibility for Taiwanese isolates to NAIs.

## Supporting Information

Table S1 **Primer pairs used in sequencing HA and NA genes.**
(PDF)

Table S2 **HA amino acid mutation statistics of 147 Taiwanese H1N1pdm viruses.**
(PDF)

Table S3 **NA amino acid mutation statistics of 147 Taiwanese H1N1pdm viruses.**
(PDF)

Table S4 **Monthly amino acid mutation statistics of 147 Taiwanese H1N1pdm viruses in HA antigenic sites, receptor binding sites, and NA antigenic sites.**
(PDF)

## References

[1] Dawood FS, Jain S, Finelli L, Shaw MW, Lindstrom S, et al (2009) Emergence of a novel swine-origin influenza A (H1N1) virus in humans. N Engl J Med 360: 2605–2615. 19423869 10.1056/NEJMoa0903810

[2] Garten RJ, Davis CT, Russell CA, Shu B, Lindstrom S, et al (2009) Antigenic and genetic characteristics of swine-origin 2009 A(H1N1) influenza viruses circulating in humans. Science 325: 197–201. 19465683 10.1126/science.1176225 PMC3250984

[3] Bright RA, Medina MJ, Xu X, Perez-Oronoz G, Wallis TR, et al (2005) Incidence of adamantane resistance among influenza A (H3N2) viruses isolated worldwide from 1994 to 2005: a cause for concern. Lancet 366: 1175–1181. 16198766 10.1016/S0140-6736(05)67338-2

[4] Deyde VM, Xu X, Bright RA, Shaw M, Smith CB, et al (2007) Surveillance of resistance to adamantanes among influenza A(H3N2) and A(H1N1) viruses isolated worldwide. J Infect Dis 196: 249–257. 17570112 10.1086/518936

[5] Renaud C, Kuypers J, Englund JA (2011) Emerging oseltamivir resistance in seasonal and pandemic influenza A/H1N1. J Clin Virol 52: 70–78. 21684202 10.1016/j.jcv.2011.05.019

[6] Smith GJ, Vijaykrishna D, Bahl J, Lycett SJ, Worobey M, et al (2009) Origins and evolutionary genomics of the 2009 swine-origin H1N1 influenza A epidemic. Nature 459: 1122–1125. 19516283 10.1038/nature08182

[7] Moscona A (2009) Global transmission of oseltamivir-resistant influenza. N Engl J Med 360: 953–956. 19258250 10.1056/NEJMp0900648

[8] Debarre F, Bonhoeffer S, Regoes RR (2007) The effect of population structure on the emergence of drug resistance during influenza pandemics. J R Soc Interface 4: 893–906. 17609176 10.1098/rsif.2007.1126 PMC2394556

[9] Deyde VM, Okomo-Adhiambo M, Sheu TG, Wallis TR, Fry A, et al (2009) Pyrosequencing as a tool to detect molecular markers of resistance to neuraminidase inhibitors in seasonal influenza A viruses. Antiviral Res 81: 16–24. 18835410 10.1016/j.antiviral.2008.08.008

[10] Igarashi M, Ito K, Yoshida R, Tomabechi D, Kida H, et al (2010) Predicting the antigenic structure of the pandemic (H1N1) 2009 influenza virus hemagglutinin. PLoS One 5: e8553. 20049332 10.1371/journal.pone.0008553 PMC2797400

[11] Yang H, Carney P, Stevens J (2010) Structure and Receptor binding properties of a pandemic H1N1 virus hemagglutinin. PLoS Curr 2: RRN1152. 20352039 10.1371/currents.RRN1152 PMC2846141

[12] Maurer-Stroh S, Ma J, Lee RT, Sirota FL, Eisenhaber F (2009) Mapping the sequence mutations of the 2009 H1N1 influenza A virus neuraminidase relative to drug and antibody binding sites. Biol Direct 4: 18; discussion 18. 10.1186/1745-6150-4-18 PMC2691737 19457254

[13] Abed Y, Baz M, Boivin G (2006) Impact of neuraminidase mutations conferring influenza resistance to neuraminidase inhibitors in the N1 and N2 genetic backgrounds. Antivir Ther 11: 971–976. 17302366

[14] de Jong MD, Tran TT, Truong HK, Vo MH, Smith GJ, et al (2005) Oseltamivir resistance during treatment of influenza A (H5N1) infection. N Engl J Med 353: 2667–2672. 16371632 10.1056/NEJMoa054512

[15] Gubareva LV, Kaiser L, Matrosovich MN, Soo-Hoo Y, Hayden FG (2001) Selection of influenza virus mutants in experimentally infected volunteers treated with oseltamivir. J Infect Dis 183: 523–531. 11170976 10.1086/318537

[16] Hurt AC, Lee RT, Leang SK, Cui L, Deng YM, et al.. (2011) Increased detection in Australia and Singapore of a novel influenza A(H1N1)2009 variant with reduced oseltamivir and zanamivir sensitivity due to a S247N neuraminidase mutation. Euro Surveill 16. 21679678

[17] Sheu TG, Deyde VM, Okomo-Adhiambo M, Garten RJ, Xu X, et al (2008) Surveillance for neuraminidase inhibitor resistance among human influenza A and B viruses circulating worldwide from 2004 to 2008. Antimicrob Agents Chemother 52: 3284–3292. 18625765 10.1128/AAC.00555-08 PMC2533500

[18] Ujike M, Ejima M, Anraku A, Shimabukuro K, Obuchi M, et al (2011) Monitoring and characterization of oseltamivir-resistant pandemic (H1N1) 2009 virus, Japan, 2009–2010. Emerg Infect Dis 17: 470–479. 21392439 10.3201/eid1703.101188 PMC3166015

[19] Pan C, Cheung B, Tan S, Li C, Li L, et al (2010) Genomic signature and mutation trend analysis of pandemic (H1N1) 2009 influenza A virus. PLoS One 5: e9549. 20221396 10.1371/journal.pone.0009549 PMC2833199

[20] Potdar VA, Chadha MS, Jadhav SM, Mullick J, Cherian SS, et al (2010) Genetic characterization of the influenza A pandemic (H1N1) 2009 virus isolates from India. PLoS One 5: e9693. 20300625 10.1371/journal.pone.0009693 PMC2837743

[21] Ilyicheva T, Susloparov I, Durymanov A, Romanovskaya A, Sharshov K, et al (2011) Influenza A/H1N1pdm virus in Russian Asia in 2009–2010. Infect Genet Evol 11: 2107–2112. 21600305 10.1016/j.meegid.2011.05.002

[22] Graham M, Liang B, Van Domselaar G, Bastien N, Beaudoin C, et al (2011) Nationwide molecular surveillance of pandemic H1N1 influenza A virus genomes: Canada, 2009. PLoS One 6: e16087. 21249207 10.1371/journal.pone.0016087 PMC3017559

[23] Furuse Y, Shimabukuro K, Odagiri T, Sawayama R, Okada T, et al (2010) Comparison of selection pressures on the HA gene of pandemic (2009) and seasonal human and swine influenza A H1 subtype viruses. Virology 405: 314–321. 20598336 10.1016/j.virol.2010.06.018

[24] Yang JR, Huang YP, Chang FY, Hsu LC, Lin YC, et al (2011) New variants and age shift to high fatality groups contribute to severe successive waves in the 2009 influenza pandemic in Taiwan. PLoS One 6: e28288. 22140569 10.1371/journal.pone.0028288 PMC3227656

