# Supplementary material for: Amino Acids Transitioning of 2009 H1N1pdm in Taiwan from 2009 to 2011
Source: PLoS One. 2012 Sep 24;7(9):e45946. doi: 10.1371/journal.pone.0045946 (PMC3454337; doi:10.1371/journal.pone.0045946)
Supplement: Table S1 — Primer pairs used in sequencing HA and NA genes. (PDF) [file pone.0045946.s001.pdf]

Supplementary Table S1 – Primer pairs used in sequencing HA and NA genes

Primer	Sequence(5'→3')	Positions	Size
[1]HA1 Forward	TGTAAAACGACGGCCAGTATACGACTAGCAAAAGCAGGGG	–41 ~ –19	943 bp
[1]HA943 Reverse	CAGGAAACAGCTATGACCGAAAKGGGAGRCTGGTGTTTA	928 ~ 908	
[1]HA736 Forward	TGTAAAACGACGGCCAGTAGRATGRACTATTACTGGAC	727 ~ 746	564 bp
HA1300 Reverse	CCAGGAAACCATCATCAAC	1300 ~ 1282	
[1]HA1204 Forward	TGTAAAACGACGGCCAGTAAGATGAAYACRCARTTCACAG	1202 ~ 1225	574 bp
[1]HA1778 Reverse	CAGGAAACAGCTATGACCGTGTCAGTAGAAACAAGGGTGTTT	1750 ~ 1727	
NA0 Forward	TTAAAATGAATCCAAACCAAAAGA	–5 ~ 19	630 bp
NA630 Reverse	TGTACTTTAACACAGCCACTGCCC	625 ~ 620	
NA536 Forward	GGTCAGCAAGCGCATGYCATGA	536 ~ 557	527 bp
NA1063 Reverse	ACCATTGCCGTATTTGAATGAAAA	1068 ~ 1045	
NA941 Forward	ATAGGATACATATGCAGTGGGA	940 ~ 961	511 bp
NA1452 Reverse	AGTAGAAACAAGGAGTTTTTT	1438 ~ 1418	

[1]Extended from WHO primers (http://www.who.int/csr/resources/publications/swineflu/sequencing_primers/en/index.html) by adding underlined nucleotides.
