# Supplementary material for: Amino Acids Transitioning of 2009 H1N1pdm in Taiwan from 2009 to 2011
Source: PLoS One. 2012 Sep 24;7(9):e45946. doi: 10.1371/journal.pone.0045946 (PMC3454337; doi:10.1371/journal.pone.0045946)
Supplement: Table S2 — HA amino acid mutation statistics of 147 Taiwanese H1N1pdm viruses. (PDF) [file pone.0045946.s002.pdf]

Supplementary Table S2 - HA amino acid mutation statistics of 147 Taiwanese H1N1pdm viruses

From	To	1st season (2009/2010)										2nd season (2010/2011)							SUM	Mutation Frequency %
		Jun	Jul	Aug	Sep	Oct	Nov	Dec	Jan	Feb	May	Aug	Sep	Oct	Nov	Dec	Jan	Feb		
**8L**	**M**													**1**	**1**			**6**	**8**	**5.44**
10Y	C							1											1	0.68
12F	L			1														1	2	1.36
13A	V				1				1	1									3	2.04
	S							1											1	0.68
**14T**	**I**																**3**	**6**	**9**	**6.12**
26A	T																	1	1	0.68
33V	I					1													1	0.68
36V	I								2				1						3	2.04
39K	R				3														3	2.04
47V	A				1														1	0.68
	F						1												1	0.68
49L	I										1								1	0.68
50L	I																	1	1	0.68
52D	N	3																	3	2.04
56G	R								1										1	0.68
64V	G										1								1	0.68
65A	V					1													1	0.68
85E	D																1	1	2	1.36
**100P**	**S**	**13**	**5**	**7**	**14**	**9**	**8**	**9**	**8**	**2**	**2**	**5**	**5**	**7**	**6**	**8**	**18**	**19**	**145**	**98.64**
	Y																	1	1	0.68
101S	N				1				1	1							1		4	2.72
**114D**	**N**																**5**	**9**	**14**	**9.52**
129E	V						1												1	0.68
137T	A																3	1	4	2.72
141P	S			1															1	0.68
**142N**	**D**										**1**	**3**	**3**	**3**	**5**	**2**	**2**		**19**	**12.93**
	S																1		1	0.68
145S	P						1					2	1	1					5	3.40

151A	T												1			1			2	1.36
155H	Y		1																1	0.68
156A	T													1	3				4	2.72
158A	S						1												1	0.68
	E																	1	1	0.68
160S	G													2		2	9	8	21	14.29
	N															2			2	1.36
172G	E													1					1	0.68
173N	T																	1	1	0.68
176P	S												2						2	1.36
177K	T								1										1	0.68
180K	T												1						1	0.68
183I	F					1													1	0.68
187G	R											1		1					2	1.36
188K	R					1	1	1											3	2.04
189E	D														2	2			4	2.72
200S	P												1			1			2	1.36
201T	N																	1	1	0.68
202S	T													3	1	5	13	13	35	23.81
203A	T								1										1	0.68
207S	I																1		1	0.68
208L	I													1					1	0.68
212A	V								1										1	0.68
214T	A	12	5	7	14	9	8	9	8	2	2	6	5	4	6	4	9	11	121	82.31
216V	A											2	1	1					4	2.72
219G	E														1	1			2	1.36
	R					1													1	0.68
	X				1														1	0.68
220S	T	12	5	6	14	8	7	9	8	2	1	6	5	7	6	8	18	18	140	95.24
222R	K			1													3	6	10	6.80
226K	M															2			2	1.36
233I	V																3	7	10	6.80

239D	G														1			1	2	**1.36**
	E				1														1	**0.68**
**266V**	**L**				**1**	**1**											**5**	**7**	**14**	**9.52**
	A									1									1	**0.68**
275E	G																	1	1	**0.68**
276R	K											1						1	2	**1.36**
280S	F								1										1	**0.68**
282I	V																	1	1	**0.68**
286D	N																1		1	**0.68**
288P	S												1						1	**0.68**
289V	I										1								1	**0.68**
294T	A									1									1	**0.68**
295T	I											1							1	**0.68**
**300K**	**E**	**1**															**2**	**6**	**9**	**6.12**
312I	V											2	1						3	**2.04**
314P	S				1														1	**0.68**
321P	S			1															1	**0.68**
328K	R											1							1	**0.68**
331L	V															1			1	**0.68**
**338I**	**V**	**13**	**5**	**7**	**13**	**9**	**8**	**9**	**8**	**2**	**2**	**6**	**5**	**7**	**6**	**6**	**18**	**19**	**143**	**97.28**
343S	Y	1																	1	**0.68**
363D	E												1						1	**0.68**
	N																	1	1	**0.68**
369H	P															1			1	**0.68**
371Q	R							1											1	**0.68**
378Y	D													1					1	**0.68**
381D	G	1		1															2	**1.36**
387N	D													1	3				4	**2.72**
	T																	1	1	**0.68**
389I	F											2	1	1					4	**2.72**
**391E**	**K**	**1**		**2**	**6**	**7**	**6**	**9**	**6**	**2**	**1**	**4**	**3**	**6**	**6**	**6**	**6**	**16**	**87**	**59.18**
	G															1			1	**0.68**
393T	I															1			1	**0.68**

422E	G															1			1	**0.68**
428V	I	1														1			2	**1.36**
451T	N													1					1	**0.68**
460K	N															2			2	**1.36**
461N	K				1	1													2	**1.36**
466V	I					1								1	3				5	**3.40**
**468S**	**N**	**1**					**1**							**3**	**1**	**5**	**13**	**13**	**37**	**25.17**
472N	T							1											1	**0.68**
477I	T			1													1		2	**1.36**
490N	D											1							1	**0.68**
491T	M											1	1						2	**1.36**
493M	I															1			1	**0.68**
495S	N											1							1	**0.68**
496V	I																	1	1	**0.68**
499G	E																2		2	**1.36**
507S	L													2					2	**1.36**
516E	K						1							1	3				5	**3.40**
521K	N				1														1	**0.68**
526R	M															1			1	**0.68**
Accumulated mutations		59	21	35	73	50	44	50	47	14	12	45	39	57	54	65	138	180	**983**	
Monthly sample count		**13**	**5**	**7**	**14**	**9**	**8**	**9**	**8**	**2**	**2**	**6**	**5**	**7**	**6**	**8**	**18**	**20**	**147**	
Mutations per sample		4.54	4.20	5.00	5.21	5.56	5.50	5.56	5.88	7.00	6.00	7.50	7.80	8.14	9.00	8.13	7.67	9.00	**6.69**	
