# Supplementary material for: Amino Acids Transitioning of 2009 H1N1pdm in Taiwan from 2009 to 2011
Source: PLoS One. 2012 Sep 24;7(9):e45946. doi: 10.1371/journal.pone.0045946 (PMC3454337; doi:10.1371/journal.pone.0045946)
Supplement: Table S3 — NA amino acid mutation statistics of 147 Taiwanese H1N1pdm viruses. (PDF) [file pone.0045946.s003.pdf]

## Supplementary Table S3 - NA amino acid mutation statistics of 147 Taiwanese H1N1pdm viruses

| From | To | 1st season (2009/2010) | | | | | | | | | | 2nd season (2010/2011) | | | | | | | SUM | Mutation Frequency % |
|---|---|---|---|---|---|---|---|---|---|---|---|---|---|---|---|---|---|---|---|---|
| | | Jun | Jul | Aug | Sep | Oct | Nov | Dec | Jan | Feb | May | Aug | Sep | Oct | Nov | Dec | Jan | Feb | | |
| 11G | G | | | | | | | | | | 1 | | | | | | | | 1 | 0.68 |
| **15M** | **I** | | | | | | | | | | **1** | **1** | **2** | **2** | **4** | | **2** | | **12** | **8.16** |
| 26I | V | | | | | | 1 | | | | | | | | | | | | 1 | 0.68 |
| 29I | V | | | | | | | | | | | | | | | 1 | | | 1 | 0.68 |
| 34I | T | | | | | | | | 1 | | | | | | | | | | 1 | 0.68 |
| 38I | L | | | | | | | | | | | | | | | | 1 | 2 | 3 | 2.04 |
| **44N** | **S** | | | | | | | | | | | | | **3** | **1** | **6** | **9** | **9** | **28** | **19.05** |
| 48T | P | | | | | | | | | | | | | | | 1 | | | 1 | 0.68 |
| 50N | D | | | | | | | | | | | | | 1 | | | | | 1 | 0.68 |
| 51Q | P | | 1 | | | | | | | | | | | | | | | | 1 | 0.68 |
| 72T | I | | | | 1 | | | | | | | | | | | | | | 1 | 0.68 |
| 74F | L | | | | | | | | | | | | 1 | | 3 | | 1 | | 5 | 3.40 |
| 76A | V | | | | | | | | | | | | | | | 1 | | | 1 | 0.68 |
| 82S | P | | | | | 1 | | | 1 | | | 1 | | | | | | | 3 | 2.04 |
| 83V | A | | | | | | | | | | | | 1 | 1 | | | 2 | | 4 | 2.72 |
| 93P | S | | | | | | | | | | | | 1 | | | | | | 1 | 0.68 |
| 105S | N | | | | | | | | | | | 1 | | | | | | | 1 | 0.68 |
| **106V** | **I** | **13** | **5** | **7** | **14** | **9** | **8** | **9** | **8** | **2** | **1** | **6** | **5** | **7** | **6** | **8** | **18** | **20** | **146** | **99.32** |
| 127L | S | | | | | | | | | | | | | | | 1 | 3 | | 4 | 2.72 |
| 143K | I | | | | | | | | | | | | | | | 1 | | | 1 | 0.68 |
| 149I | F | | | | | | | | | | | 1 | | | | | | | 1 | 0.68 |
| 153S | T | | | | | 1 | | | | | | | | | | | | | 1 | 0.68 |
| 166V | I | | | | 1 | | | | | | | | | | | | | | 1 | 0.68 |
| 188I | V | | | | | | | 1 | | | | | | | | | | | 1 | 0.68 |
| **189N** | **S** | | | | | | | | | | | **1** | **3** | **2** | **4** | | **2** | | **12** | **8.16** |
| 203V | M | | | | | | 1 | | | | | | | | | | | | 1 | 0.68 |
| 211I | V | | | | 3 | | | | | | | | | | | | | | 3 | 2.04 |
| 222N | K | 1 | | | | | | | | | | | | | | | | | 1 | 0.68 |
| | S | | | 1 | | | | | | | | | | | | | | | 1 | 0.68 |
| 223I | T | | | | | | | | 1 | | | | | | | | | | 1 | 0.68 |
| 229S | F | | | | | | | | | | | | | | | | | 1 | 1 | 0.68 |
| 237S | F | | | | | | | | | | | | | | | | | 2 | 2 | 1.36 |

| | | | | | | | | | | | | | | | | | | | | |
|---|---|---|---|---|---|---|---|---|---|---|---|---|---|---|---|---|---|---|---|---|
| 240T | I | | | | | | | | | | | | | | | | 2 | | 2 | 1.36 |
| **241V** | **I** | | | | | | | | | | | | | **3** | **1** | **7** | **13** | **12** | **36** | **24.49** |
| | M | | | | | | | | | | | | | | | | | 1 | 1 | 0.68 |
| 247S | N | | | | | | | | | | | | | | | 1 | | | 1 | 0.68 |
| **248N** | **D** | **13** | **5** | **7** | **14** | **9** | **8** | **9** | **8** | **2** | **2** | **6** | **5** | **7** | **6** | **8** | **18** | **20** | **147** | **100.00** |
| 249G | R | | | | | | | | | | | | | | | | | 1 | 1 | 0.68 |
| 254K | R | | | | | | | 1 | | | | | | | | | | | 1 | 0.68 |
| 257R | K | | | | | | | | | | | | 1 | | | | | | 1 | 0.68 |
| 275H | Y | | | | | | | | | | | | | | | | | 1 | 1 | 0.68 |
| 295N | K | | | | | | | | | | | | | | | | 2 | | 2 | 1.36 |
| **299S** | **A** | | | | | | | | | | | | | | | | **3** | **7** | **10** | **6.80** |
| 308Q | H | | | | | | | | | | | | | 1 | 3 | | | | 4 | 2.72 |
| 309N | D | | | | | | | | | | | | | | | | | 1 | 1 | 0.68 |
| 311E | D | | | | | | | | | | | | | | | 2 | | | 2 | 1.36 |
| 313Q | R | | | | | | | | | | | | 1 | | | | | | 1 | 0.68 |
| 331K | R | | | | | | | | | | | | | | | 1 | 1 | | 2 | 1.36 |
| 339S | P | | | | | | | | | | | | | | | | | 1 | 1 | 0.68 |
| 354G | D | | | | | | | | | | | | | | | | | 1 | 1 | 0.68 |
| **365I** | **T** | | | | | | | | | | | **2** | **1** | **2** | **3** | | | | **8** | **5.44** |
| | F | | | | | | | | | | | | | | | | 1 | | 1 | 0.68 |
| **369N** | **K** | | | | | | | | | | | | | **3** | **1** | **7** | **13** | **13** | **37** | **25.17** |
| **374I** | **V** | | | | | | | | | | | | | | | | **3** | **7** | **10** | **6.80** |
| 375W | C | 1 | | | | | | | | | | | | | | | | | 1 | 0.68 |
| 381T | N | | | 2 | | | | | | | | | | | | | | | 2 | 1.36 |
| 386N | S | | | | | | | | | | | 2 | 1 | 1 | | | | | 4 | 2.72 |
| | K | | | | | | | | | | 1 | | | | 1 | | | | 2 | 1.36 |
| 389I | V | | | | | | | | | | | | | | 1 | 1 | | | 2 | 1.36 |
| 394V | I | | | | | | | | 2 | | | | 1 | | 1 | 1 | | | 5 | 3.40 |
| 397N | K | | | | | | | | | | 1 | | | | | | | | 1 | 0.68 |
| | I | | | | | | | | | | | | | | | | 1 | | 1 | 0.68 |
| 404G | R | 1 | | | | | | | | | | | | | | | | | 1 | 0.68 |
| 415L | M | | | | | | | | | | | | | | | | | 1 | 1 | 0.68 |
| 416D | N | | | 2 | | | | | | | | | | | | | | | 2 | 1.36 |
| 430R | Q | | | | | | | | 1 | | | | | | | | | | 1 | 0.68 |
| 436I | V | | | | | | | | | | | | 1 | | | | | | 1 | 0.68 |

| | | | | | | | | | | | | | | | | | | | | |
|---|---|---|---|---|---|---|---|---|---|---|---|---|---|---|---|---|---|---|---|---|
| 438T | I | | | | | | | | | | | | | 1 | | | | | 1 | 0.68 |
| 439S | G | | | | | | | | | | | | | | | | | 1 | 1 | 0.68 |
| 442S | I | | | | | | 2 | 2 | | | | | | | | | | | 4 | 2.72 |
| 448V | I | | | | | 1 | | | | | | | | | | | | | 1 | 0.68 |
| 454G | S | | | | | | | | | | | | | | | 1 | 1 | | 2 | 1.36 |
| | V | | | | | | | | | | | | | | | | 1 | | 1 | 0.68 |
| 462E | K | | | | | | | | | 1 | | | | | | | | | 1 | 0.68 |
| 466T | I | | | | | | | | | | | | | 1 | | | | | 1 | 0.68 |
| Accumulated mutations | | 29 | 11 | 19 | 33 | 21 | 20 | 22 | 22 | 5 | 7 | 21 | 24 | 35 | 35 | 48 | 97 | 101 | **550** | |
| Monthly sample count | | **13** | **5** | **7** | **14** | **9** | **8** | **9** | **8** | **2** | **2** | **6** | **5** | **7** | **6** | **8** | **18** | **20** | **147** | |
| Mutations per sample | | 2.23 | 2.20 | 2.71 | 2.36 | 2.33 | 2.50 | 2.44 | 2.75 | 2.50 | 3.50 | 3.50 | 4.80 | 5.00 | 5.83 | 6.00 | 5.39 | 5.05 | **3.74** | |
