# Supplementary material for: Amino Acids Transitioning of 2009 H1N1pdm in Taiwan from 2009 to 2011
Source: PLoS One. 2012 Sep 24;7(9):e45946. doi: 10.1371/journal.pone.0045946 (PMC3454337; doi:10.1371/journal.pone.0045946)
Supplement: Table S4 — Monthly amino acid mutation statistics of 147 Taiwanese H1N1pdm viruses in HA antigenic sites, receptor binding sites, and NA antigenic sites. (PDF) [file pone.0045946.s004.pdf]

Supplementary Table S4 - Monthly amino acid mutation statistics of 147 Taiwanese H1N1pdm viruses in HA antigenic sites, receptor binding sites, and NA antigenic sites.

(In each table, row #1 and #2 are the amino acids of A/California/04/2009, and column #2 lists the monthly virus counts. )

**(1) HA amino acid substitutions in antigenic site Sa**

| | | 141 | 142 | | 170 | 171 | 172 | 173 | 174 | | 176 | 177 | 178 | 179 | 180 | 182 |
|---|---|---|---|---|---|---|---|---|---|---|---|---|---|---|---|---|
| CA2009 | | P | N | – | K | K | G | N | S | – | P | K | L | S | K | S |
| Jun 2009 | 13 | | | | | | | | | | | | | | | |
| Jul | 5 | | | | | | | | | | | | | | | |
| Aug | 7 | S(1) | | | | | | | | | | | | | | |
| Sept | 14 | | | | | | | | | | | | | | | |
| Oct | 9 | | | | | | | | | | | | | | | |
| Nov | 8 | | | | | | | | | | | | | | | |
| Dec | 9 | | | | | | | | | | | | | | | |
| Jan 2010 | 8 | | | | | | | | | | | T(1) | | | | |
| Feb | 2 | | | | | | | | | | | | | | | |
| May | 2 | | D(1) | | | | | | | | | | | | | |
| Aug | 6 | | D(3) | | | | | | | | | | | | | |
| Sept | 5 | | D(3) | | | | | | | | S(2) | | | | T(1) | |
| Oct | 7 | | D(2) | | | | E(1) | | | | | | | | | |
| Nov | 6 | | D(5) | | | | | | | | | | | | | |
| Dec | 8 | | D(2) | | | | | | | | | | | | | |
| Jan 2011 | 18 | | D(2), S(1) | | | | | | | | | | | | | |
| Feb | 20 | | | | | | | T(1) | | | | | | | | |

**(2) HA amino acid substitutions in antigenic site Sb**

| | | 201 | 202 | 203 | 204 | 205 | 206 | 207 | 208 | 209 | 210 | 211 | 212 |
|---|---|---|---|---|---|---|---|---|---|---|---|---|---|
| CA2009 | | T | S | A | D | Q | Q | S | L | Y | Q | N | A |
| Jun 2009 | 13 | | | | | | | | | | | | |
| Jul | 5 | | | | | | | | | | | | |
| Aug | 7 | | | | | | | | | | | | |
| Sept | 14 | | | | | | | | | | | | |
| Oct | 9 | | | | | | | | | | | | |
| Nov | 8 | | | | | | | | | | | | |
| Dec | 9 | | | | | | | | | | | | |
| Jan 2010 | 8 | | | T(1) | | | | | | | | | V(1) |
| Feb | 2 | | | | | | | | | | | | |
| May | 2 | | | | | | | | | | | | |
| Aug | 6 | | | | | | | | | | | | |
| Sept | 5 | | | | | | | | | | | | |
| Oct | 7 | | T(3) | | | | | | I(1) | | | | |
| Nov | 6 | | T(1) | | | | | | | | | | |
| Dec | 8 | | T(5) | | | | | | | | | | |
| Jan 2011 | 18 | | T(13) | | | | | I(1) | | | | | |
| Feb | 20 | N(1) | T(13) | | | | | | | | | | |

**(3) HA amino acid substitutions in antigenic site Ca**

| | | 154 | 155 | 156 | 157 | 158 | 159 | | 183 | 184 | 185 | 186 | 187 | | 220 | 221 | 222 | | 238 | 239 | | 252 | 253 | 254 |
|---|---|---|---|---|---|---|---|---|---|---|---|---|---|---|---|---|---|---|---|---|---|---|---|---|
| CA2009 | | P | H | A | G | A | K | – | I | N | D | K | G | – | S | S | R | – | R | D | – | E | P | G |
| Jun 2009 | 13 | | | | | | | | | | | | | | T(12) | | | | | | | | | |
| Jul | 5 | | Y(1) | | | | | | | | | | | | T(5) | | | | | | | | | |
| Aug | 7 | | | | | | | | | | | | | | T(6) | | K(1) | | | | | | | |
| Sept | 14 | | | | | | | | | | | | | | T(14) | | | | | E(1) | | | | |
| Oct | 9 | | | | | | | | F(1) | | | | | | T(8) | | | | | | | | | |

| | | | | | | | | | | | | | | | | | | | | | | | | |
|---|---|---|---|---|---|---|---|---|---|---|---|---|---|---|---|---|---|---|---|---|---|---|---|---|
| Nov | 8 | | | | | S(1) | | | | | | | | | T(7) | | | | | | | | | |
| Dec | 9 | | | | | | | | | | | | | | T(9) | | | | | | | | | |
| Jan 2010 | 8 | | | | | | | | | | | | | | T(8) | | | | | | | | | |
| Feb | 2 | | | | | | | | | | | | | | T(2) | | | | | | | | | |
| May | 2 | | | | | | | | | | | | | | T(1) | | | | | | | | | |
| Aug | 6 | | | | | | | | | | | | R(1) | | T(6) | | | | | | | | | |
| Sept | 5 | | | | | | | | | | | | | | T(5) | | | | | | | | | |
| Oct | 7 | | | T(1) | | | | | | | | | R(1) | | T(7) | | | | | | | | | |
| Nov | 6 | | | T(3) | | | | | | | | | | | T(6) | | | | | G(1) | | | | |
| Dec | 8 | | | | | | | | | | | | | | T(8) | | | | | | | | | |
| Jan 2011 | 18 | | | | | | | | | | | | | | T(18) | | K(3) | | | | | | | |
| Feb | 20 | | | | | E(1) | | | | | | | | | T(18) | | K(6) | | | G(1) | | | | |

| (4) HA amino acid substitutions in antigenic site Cb | | | | | | | |
|---|---|---|---|---|---|---|---|
| | | 87 | 88 | 89 | 90 | 91 | 92 |
| CA2009 | | L | S | T | A | S | S |
| Jun 2009 | 13 | | | | | | |
| Jul | 5 | | | | | | |
| Aug | 7 | | | | | | |
| Sept | 14 | | | | | | |
| Oct | 9 | | | | | | |
| Nov | 8 | | | | | | |
| Dec | 9 | | | | | | |
| Jan 2010 | 8 | | | | | | |
| Feb | 2 | | | | | | |
| May | 2 | | | | | | |
| Aug | 6 | | | | | | |
| Sept | 5 | | | | | | |
| Oct | 7 | | | | | | |
| Nov | 6 | | | | | | |
| Dec | 8 | | | | | | |
| Jan 2011 | 18 | | | | | | |
| Feb | 20 | | | | | | |

| (5) HA amino acid substitutions in receptor binding sites (RBS) | | | | | | | | | | | | | | | | | | | | | | | | |
|---|---|---|---|---|---|---|---|---|---|---|---|---|---|---|---|---|---|---|---|---|---|---|---|---|
| | | 148 | 149 | 150 | 151 | 152 | | 201 | 202 | 203 | 204 | 205 | 206 | 207 | 208 | | 235 | 236 | 237 | 238 | 239 | 240 | 241 | 242 |
| CA2009 | | G | V | T | A | A | | T | S | A | D | Q | Q | S | L | – | P | K | V | R | D | Q | E | G |
| Jun 2009 | 13 | | | | | | | | | | | | | | | | | | | | | | | |
| Jul | 5 | | | | | | | | | | | | | | | | | | | | | | | |
| Aug | 7 | | | | | | | | | | | | | | | | | | | | | | | |
| Sept | 14 | | | | | | | | | | | | | | | | | | | | E(1) | | | |
| Oct | 9 | | | | | | | | | | | | | | | | | | | | | | | |
| Nov | 8 | | | | | | | | | | | | | | | | | | | | | | | |
| Dec | 9 | | | | | | | | | | | | | | | | | | | | | | | |
| Jan 2010 | 8 | | | | | | | | | T(1) | | | | | | | | | | | | | | |
| Feb | 2 | | | | | | | | | | | | | | | | | | | | | | | |
| May | 2 | | | | | | | | | | | | | | | | | | | | | | | |
| Aug | 6 | | | | | | | | | | | | | | | | | | | | | | | |
| Sept | 5 | | | | T(1) | | | | | | | | | | | | | | | | | | | |
| Oct | 7 | | | | | | | | T(3) | | | | | | I(1) | | | | | | | | | |
| Nov | 6 | | | | | | | | T(1) | | | | | | | | | | | | G(1) | | | |
| Dec | 8 | | | | T(1) | | | | T(5) | | | | | | | | | | | | | | | |
| Jan 2011 | 18 | | | | | | | | T(13) | | | | | I(1) | | | | | | | | | | |
| Feb | 20 | | | | | | | N(1 | T(13) | | | | | | | | | | | | G(1) | | | |

**(6) NA amino acid substitutions in antigenic sites - part 1**

| | | 83 | 84 | 85 | 86 | 87 | 88 | 89 | 90 | 91 | 92 | 93 | 94 | 95 | 96 | 97 | 98 | 99 |
|---|---|---|---|---|---|---|---|---|---|---|---|---|---|---|---|---|---|---|
| CA2009 | | V | K | I | A | G | N | S | S | L | C | P | V | S | G | W | A | I |
| Jun 2009 | 13 | | | | | | | | | | | | | | | | | |
| Jul | 5 | | | | | | | | | | | | | | | | | |
| Aug | 7 | | | | | | | | | | | | | | | | | |
| Sept | 14 | | | | | | | | | | | | | | | | | |
| Oct | 9 | | | | | | | | | | | | | | | | | |
| Nov | 8 | | | | | | | | | | | | | | | | | |
| Dec | 9 | | | | | | | | | | | | | | | | | |
| Jan 2010 | 8 | | | | | | | | | | | | | | | | | |
| Feb | 2 | | | | | | | | | | | | | | | | | |
| May | 2 | | | | | | | | | | | | | | | | | |
| Aug | 6 | | | | | | | | | | | | | | | | | |
| Sept | 5 | A(1) | | | | | | | | | | S(1) | | | | | | |
| Oct | 7 | A(1) | | | | | | | | | | | | | | | | |
| Nov | 6 | | | | | | | | | | | | | | | | | |
| Dec | 8 | | | | | | | | | | | | | | | | | |
| Jan 2011 | 18 | A(2) | | | | | | | | | | | | | | | | |
| Feb | 20 | | | | | | | | | | | | | | | | | |

**(6) NA amino acid substitutions in antigenic sites - part 2**

| | | 103 | 104 | 105 | 106 | 107 | 108 | 109 | 110 | 111 | 112 | 113 | 114 | 115 | 116 | 117 | 118 | 119 | 120 | 121 | 122 | 123 | 124 | 125 | 126 | 127 | 128 | 129 | 130 | 131 | 132 | 133 | 134 | 135 | 136 | 137 | 138 | 139 | 140 | 141 | 142 | 143 | 144 |
|---|---|---|---|---|---|---|---|---|---|---|---|---|---|---|---|---|---|---|---|---|---|---|---|---|---|---|---|---|---|---|---|---|---|---|---|---|---|---|---|---|---|---|---|
| CA2009 | | D | N | S | V | R | I | G | S | K | G | D | V | F | V | I | R | E | P | F | I | S | C | S | P | L | E | C | R | T | F | F | L | T | Q | G | A | L | L | N | D | K | H |
| Jun 2009 | 13 | | | | I(13) | | | | | | | | | | | | | | | | | | | | | | | | | | | | | | | | | | | | | | |
| Jul | 5 | | | | I(5) | | | | | | | | | | | | | | | | | | | | | | | | | | | | | | | | | | | | | | |
| Aug | 7 | | | | I(7) | | | | | | | | | | | | | | | | | | | | | | | | | | | | | | | | | | | | | | |
| Sept | 14 | | | | I(14) | | | | | | | | | | | | | | | | | | | | | | | | | | | | | | | | | | | | | | |
| Oct | 9 | | | | I(9) | | | | | | | | | | | | | | | | | | | | | | | | | | | | | | | | | | | | | | |
| Nov | 8 | | | | I(8) | | | | | | | | | | | | | | | | | | | | | | | | | | | | | | | | | | | | | | |
| Dec | 9 | | | | I(9) | | | | | | | | | | | | | | | | | | | | | | | | | | | | | | | | | | | | | | |
| Jan 2010 | 8 | | | | I(8) | | | | | | | | | | | | | | | | | | | | | | | | | | | | | | | | | | | | | | |
| Feb | 2 | | | | I(2) | | | | | | | | | | | | | | | | | | | | | | | | | | | | | | | | | | | | | | |
| May | 2 | | | | I(1) | | | | | | | | | | | | | | | | | | | | | | | | | | | | | | | | | | | | | | |
| Aug | 6 | | | N(1 | I(6) | | | | | | | | | | | | | | | | | | | | | | | | | | | | | | | | | | | | | | |
| Sept | 5 | | | | I(5) | | | | | | | | | | | | | | | | | | | | | | | | | | | | | | | | | | | | | | |
| Oct | 7 | | | | I(7) | | | | | | | | | | | | | | | | | | | | | | | | | | | | | | | | | | | | | | |
| Nov | 6 | | | | I(6) | | | | | | | | | | | | | | | | | | | | | | | | | | | | | | | | | | | | | | |
| Dec | 8 | | | | I(8) | | | | | | | | | | | | | | | | | | | | | S(1) | | | | | | | | | | | | | | | | I(1) | |
| Jan 2011 | 18 | | | | I(18) | | | | | | | | | | | | | | | | | | | | | S(3) | | | | | | | | | | | | | | | | | |
| Feb | 20 | | | | I(20) | | | | | | | | | | | | | | | | | | | | | | | | | | | | | | | | | | | | | | |

**(6) NA amino acid substitutions in antigenic sites - part 3**

| | | 156 | 157 | 158 | 159 | 160 | 161 | 162 | 163 | 164 | 165 | 166 | 167 | 168 | 169 | 170 | 171 | 172 | 173 | 174 | 175 | 176 | 177 | 178 | 179 | 180 | 181 | 182 | 183 | 184 | 185 | 186 | 187 | 188 | 189 | 190 |
|---|---|---|---|---|---|---|---|---|---|---|---|---|---|---|---|---|---|---|---|---|---|---|---|---|---|---|---|---|---|---|---|---|---|---|---|---|
| CA2009 | | R | T | L | M | S | C | P | I | G | E | V | P | S | P | Y | N | S | R | F | E | S | V | A | W | S | A | S | A | C | H | D | G | I | N | W |
| Jun 2009 | 13 | | | | | | | | | | | | | | | | | | | | | | | | | | | | | | | | | | | |
| Jul | 5 | | | | | | | | | | | | | | | | | | | | | | | | | | | | | | | | | | | |
| Aug | 7 | | | | | | | | | | | | | | | | | | | | | | | | | | | | | | | | | | | |
| Sept | 14 | | | | | | | | | | | I(1) | | | | | | | | | | | | | | | | | | | | | | | | |
| Oct | 9 | | | | | | | | | | | | | | | | | | | | | | | | | | | | | | | | | | | |
| Nov | 8 | | | | | | | | | | | | | | | | | | | | | | | | | | | | | | | | | | | |
| Dec | 9 | | | | | | | | | | | | | | | | | | | | | | | | | | | | | | | | | V(1) | | |
| Jan 2010 | 8 | | | | | | | | | | | | | | | | | | | | | | | | | | | | | | | | | | | |
| Feb | 2 | | | | | | | | | | | | | | | | | | | | | | | | | | | | | | | | | | | |
| May | 2 | | | | | | | | | | | | | | | | | | | | | | | | | | | | | | | | | | | |

| | | | | | | | | | | | | | | | | | | | | | | | | | | | | | | | | | | | | |
|---|---|---|---|---|---|---|---|---|---|---|---|---|---|---|---|---|---|---|---|---|---|---|---|---|---|---|---|---|---|---|---|---|---|---|---|---|
| Aug | 6 | | | | | | | | | | | | | | | | | | | | | | | | | | | | | | | | | S(1) | |
| Sept | 5 | | | | | | | | | | | | | | | | | | | | | | | | | | | | | | | | | S(3) | |
| Oct | 7 | | | | | | | | | | | | | | | | | | | | | | | | | | | | | | | | | S(2) | |
| Nov | 6 | | | | | | | | | | | | | | | | | | | | | | | | | | | | | | | | | S(4) | |
| Dec | 8 | | | | | | | | | | | | | | | | | | | | | | | | | | | | | | | | | | |
| Jan 2011 | 18 | | | | | | | | | | | | | | | | | | | | | | | | | | | | | | | | | S(2) | |
| Feb | 20 | | | | | | | | | | | | | | | | | | | | | | | | | | | | | | | | | | |

**(6) NA amino acid substitutions in antigenic sites - part 4**

| | | 252 | 253 | 254 | 255 | 256 | 257 | 258 | 259 | 260 | 261 | 262 | 263 | 264 | 265 | 266 | 267 | 268 | 269 | 270 | 271 | 272 | 273 | 274 | 275 | 276 | 277 | 278 | 279 | 280 | 281 | 282 | 283 | 284 | 285 | 286 | 287 | 288 | 289 | 290 | 291 | 292 | 293 | 294 | 295 | 296 | 297 | 298 | 299 | 300 | 301 | 302 | 303 |
|---|---|---|---|---|---|---|---|---|---|---|---|---|---|---|---|---|---|---|---|---|---|---|---|---|---|---|---|---|---|---|---|---|---|---|---|---|---|---|---|---|---|---|---|---|---|---|---|---|---|---|---|---|---|
| CA2009 | | S | Y | K | I | F | R | I | E | K | G | K | I | V | K | S | V | E | M | N | A | P | N | Y | H | Y | E | E | C | S | C | Y | P | D | S | S | E | I | T | C | V | C | R | D | N | W | H | G | S | N | R | P | W |
| Jun 2009 | 13 | | | | | | | | | | | | | | | | | | | | | | | | | | | | | | | | | | | | | | | | | | | | | | | | | | | | |
| Jul | 5 | | | | | | | | | | | | | | | | | | | | | | | | | | | | | | | | | | | | | | | | | | | | | | | | | | | | |
| Aug | 7 | | | | | | | | | | | | | | | | | | | | | | | | | | | | | | | | | | | | | | | | | | | | | | | | | | | | |
| Sept | 14 | | | | | | | | | | | | | | | | | | | | | | | | | | | | | | | | | | | | | | | | | | | | | | | | | | | | |
| Oct | 9 | | | | | | | | | | | | | | | | | | | | | | | | | | | | | | | | | | | | | | | | | | | | | | | | | | | | |
| Nov | 8 | | | | | | | | | | | | | | | | | | | | | | | | | | | | | | | | | | | | | | | | | | | | | | | | | | | | |
| Dec | 9 | | | R(1) | | | | | | | | | | | | | | | | | | | | | | | | | | | | | | | | | | | | | | | | | | | | | | | | | |
| Jan 2010 | 8 | | | | | | | | | | | | | | | | | | | | | | | | | | | | | | | | | | | | | | | | | | | | | | | | | | | | |
| Feb | 2 | | | | | | | | | | | | | | | | | | | | | | | | | | | | | | | | | | | | | | | | | | | | | | | | | | | | |
| May | 2 | | | | | | | | | | | | | | | | | | | | | | | | | | | | | | | | | | | | | | | | | | | | | | | | | | | | |
| Aug | 6 | | | | | | | | | | | | | | | | | | | | | | | | | | | | | | | | | | | | | | | | | | | | | | | | | | | | |
| Sept | 5 | | | | | | K(1) | | | | | | | | | | | | | | | | | | | | | | | | | | | | | | | | | | | | | | | | | | | | | | |
| Oct | 7 | | | | | | | | | | | | | | | | | | | | | | | | | | | | | | | | | | | | | | | | | | | | | | | | | | | | |
| Nov | 6 | | | | | | | | | | | | | | | | | | | | | | | | | | | | | | | | | | | | | | | | | | | | | | | | | | | | |
| Dec | 8 | | | | | | | | | | | | | | | | | | | | | | | | | | | | | | | | | | | | | | | | | | | | | | | | | | | | |
| Jan 2011 | 18 | | | | | | | | | | | | | | | | | | | | | | | | | | | | | | | | | | | | | | | | | | | | K(1) | | | | A(3) | | | | |
| Feb | 20 | | | | | | | | | | | | | | | | | | | | | | | | Y(1) | | | | | | | | | | | | | | | | | | | | | | | | A(7) | | | | |

**(6) NA amino acid substitutions in antigenic sites - part 5**

| | | 330 | | 332 | | 340 | 341 | 342 | 343 | 344 | 345 | | 368 | | 370 | | 386 | 387 | 388 | 389 | 390 | 391 | 392 | 393 | 394 | 395 |
|---|---|---|---|---|---|---|---|---|---|---|---|---|---|---|---|---|---|---|---|---|---|---|---|---|---|---|
| CA2009 | | D | – | T | – | S | N | G | A | N | G | – | R | – | G | – | N | F | S | I | K | Q | D | I | V | G |
| Jun 2009 | 13 | | | | | | | | | | | | | | | | | | | | | | | | | |
| Jul | 5 | | | | | | | | | | | | | | | | | | | | | | | | | |
| Aug | 7 | | | | | | | | | | | | | | | | | | | | | | | | | |
| Sept | 14 | | | | | | | | | | | | | | | | | | | | | | | | | |
| Oct | 9 | | | | | | | | | | | | | | | | | | | | | | | | | |
| Nov | 8 | | | | | | | | | | | | | | | | | | | | | | | | | |
| Dec | 9 | | | | | | | | | | | | | | | | | | | | | | | | | |
| Jan 2010 | 8 | | | | | | | | | | | | | | | | | | | | | | | | I(2) | |
| Feb | 2 | | | | | | | | | | | | | | | | | | | | | | | | | |
| May | 2 | | | | | | | | | | | | | | | | K(1) | | | | | | | | | |
| Aug | 6 | | | | | | | | | | | | | | | | S(2) | | | | | | | | | |
| Sept | 5 | | | | | | | | | | | | | | | | S(1) | | | | | | | | I(1) | |
| Oct | 7 | | | | | | | | | | | | | | | | S(1) | | | | | | | | | |
| Nov | 6 | | | | | | | | | | | | | | | | K(1) | | | | | | | | I(1) | |
| Dec | 8 | | | | | | | | | | | | | | | | | | | V(1) | | | | | I(1) | |
| Jan 2011 | 18 | | | | | | | | | | | | | | | | | | | V(1) | | | | | | |
| Feb | 20 | | | | | | | | | | | | | | | | | | | | | | | | | |

**(6) NA amino acid substitutions in antigenic sites - part 6**

| | | 400 | | 431 | 432 | 433 | 434 | 435 | | 448 | 449 | 450 | 451 | 452 | 453 | 454 | 455 | 456 | 457 | 458 | 459 | 460 | 461 | 462 | 463 | 464 | 465 | 466 | 467 | 468 |
|---|---|---|---|---|---|---|---|---|---|---|---|---|---|---|---|---|---|---|---|---|---|---|---|---|---|---|---|---|---|---|
| CA2009 | | S | – | P | K | E | N | T | – | V | N | S | D | T | V | G | W | S | W | P | D | G | A | E | L | P | F | T | I | D |
| Jun 2009 | 13 | | | | | | | | | | | | | | | | | | | | | | | | | | | | | |
| Jul | 5 | | | | | | | | | | | | | | | | | | | | | | | | | | | | | |

| | | | | | | | | | | | | | | | | | | | | | | | | | | | | | | |
|---|---|---|---|---|---|---|---|---|---|---|---|---|---|---|---|---|---|---|---|---|---|---|---|---|---|---|---|---|---|---|
| Aug | 7 | | | | | | | | | | | | | | | | | | | | | | | | | | | | | |
| Sept | 14 | | | | | | | | | | | | | | | | | | | | | | | | | | | | | |
| Oct | 9 | | | | | | | | | I(1) | | | | | | | | | | | | | | | | | | | | |
| Nov | 8 | | | | | | | | | | | | | | | | | | | | | | | | | | | | | |
| Dec | 9 | | | | | | | | | | | | | | | | | | | | | | | | | | | | | |
| Jan 2010 | 8 | | | | | | | | | | | | | | | | | | | | | | | | | | | | | |
| Feb | 2 | | | | | | | | | | | | | | | | | | | | | | | K(1) | | | | | | |
| May | 2 | | | | | | | | | | | | | | | | | | | | | | | | | | | | | |
| Aug | 6 | | | | | | | | | | | | | | | | | | | | | | | | | | | | | |
| Sept | 5 | | | | | | | | | | | | | | | | | | | | | | | | | | | | | |
| Oct | 7 | | | | | | | | | | | | | | | | | | | | | | | | | | | I(1) | | |
| Nov | 6 | | | | | | | | | | | | | | | | | | | | | | | | | | | | | |
| Dec | 8 | | | | | | | | | | | | | | | S(1) | | | | | | | | | | | | | | |
| Jan 2011 | 18 | | | | | | | | | | | | | | | S(1), V(1) | | | | | | | | | | | | | | |
| Feb | 20 | | | | | | | | | | | | | | | | | | | | | | | | | | | | | |
